# Development and Validation of an Up-to-Date Highly Sensitive UHPLC-MS/MS Method for the Simultaneous Quantification of Current Anti-HIV Nucleoside Analogues in Human Plasma

**DOI:** 10.3390/ph14050460

**Published:** 2021-05-13

**Authors:** Amedeo De Nicolò, Alessandra Manca, Alice Ianniello, Alice Palermiti, Andrea Calcagno, Micol Ferrara, Miriam Antonucci, Jessica Cusato, Valeria Avataneo, Elisa De Vivo, Stefano Bonora, Francesco Giuseppe De Rosa, Giovanni Di Perri, Antonio D’Avolio

**Affiliations:** 1 Department of Medical Sciences, University of Turin, Laboratory of Clinical Pharmacology and Pharmacogenetics, Amedeo di Savoia Hospital, 10149 Turin, Italy; alessandra.manca@unito.it (A.M.); alice.ianniello89@gmail.com (A.I.); alice.palermiti@gmail.com (A.P.); miriam.antonucci20@gmail.com (M.A.); jessica.cusato@unito.it (J.C.); valeria.avataneo@unito.it (V.A.); elisa.devivo59@gmail.com (E.D.V.); antonio.davolio@unito.it (A.D.); 2 Department of Medical Sciences, Unit of Infectious Diseases, University of Turin, 10149 Turin, Italy; andrea.calcagno@unito.it (A.C.); micol.ferrara29@gmail.com (M.F.); stefano.bonora@unito.it (S.B.); francescogiuseppe.derosa@unito.it (F.G.D.R.); giovanni.diperri@unito.it (G.D.P.)

**Keywords:** liquid chromatography, tandem mass spectrometry, tenofovir alafenamide, nucleoside analogues, NRTIs

## Abstract

Therapeutic options to treat HIV infection have widened in the past years, improving both effectiveness and tolerability, but nucleoside reverse transcriptase inhibitors (NRTIs) are still considered the standard backbone of the combination regimens. Therapeutic drug monitoring (TDM) can be useful for these drugs, due to concentration–effect relationship, with risk of ineffectiveness, toxicity or adherence concerns: in this scenario, robust and multiplexed methods are needed for an effective TDM activity. In this work, the first validated ultra-high spectrometry liquid chromatography coupled with tandem mass spectrometry (UHPLC-MS/MS) method is described for the high-sensitive simultaneous quantification of all the currently used NRTIs in human plasma, including tenofovir alafenamide (TAF), following FDA and EMA guidelines. The automated sample preparation consisted in the addition of an internal standard (IS) working solution, containing stable-isotope-linked drugs, protein precipitation and drying. Dry extracts were reconstituted with water, then, these underwent reversed phase chromatographic separation: compounds were detected through electrospray ionization and multiple reaction monitoring. Accuracy, precision, recovery and IS-normalized matrix effect fulfilled guidelines’ requirements. The application of this method on samples from people living with HIV (PLWH) showed satisfactory performance, being capable of quantifying the very low concentrations of tenofovir (TFV) in patients treated with TAF.

## 1. Introduction

The current application of combined antiretroviral therapy (cART) is steadily improving the treatment management for PLWH [1,2]. Despite this improvement, cART remains a life-long therapy, due to viral latency and the presence of pharmacological sanctuaries [3,4,5]. Combined ART includes in the vast majority of cases a “backbone”, which is commonly represented by two nucleoside reverse transcriptase inhibitors (NRTIs) and a highly potent “third drug” from another class: non-nucleoside reverse transcriptase inhibitors (NNRTIs), protease inhibitors (PIs), entry inhibitors (EI), fusion inhibitors (FI) and integrase inhibitors (INIs) [2]. Despite the considerable improvements over time in the effectiveness and tolerability of the cART, some concerns are still present, mainly related to the chronic nature of this therapy and, most often, associated with the use of NRTIs [6,7,8].

In detail, NRTIs are capable to exhibit mitochondrial toxicity, potentially leading to adverse events involving heart, kidney and bones [7]. Particularly, high concentrations of TFV in plasma, on a long period, have been associated with progressive renal toxicity (tubular dysfunction) and a related loss in bone mineral density [9,10,11]. For these reasons, tenofovir alafenamide (TAF) has been approved as a new TFV prodrug, designed to be converted to TFV (and its active phosphorylated metabolites) within target cells [12,13]. In fact, the administration of TAF is associated to improved pharmacokinetic (PK) features, with a 10-fold reduction in the plasma concentrations of TFV, compared with the previous prodrug (tenofovir disoproxyl fumarate, TDF), but considerably higher concentrations in peripheral blood mononuclear cells (PBMCs) [12]. These favorable features increased the interest for the use of TAF also for treatment against HBV for chronic hepatitis B [14].

On the other hand, the long-term treatment and the potential presence of comorbidities and concomitant treatments could significantly reduce patients’ compliance, thus reducing the overall exposure to the drugs and, perhaps, treatment effectiveness [15,16,17].

In this scenario, therapeutic drug monitoring (TDM) has already been established as a useful tool to optimize the posology and to identify cases of poor adherence, finally resulting in increased effectiveness and less toxicity [6,9,10,18,19,20,21]. Despite this, the TDM activity for NRTIs is still not widely performed, mainly because of the lack of generally acknowledged therapeutic ranges, but also because of the lack of reliable, validated, but still economical, multiplexed analytical methods. In fact, several methods have already been described for the quantification of NRTIs, but these do not cover the whole panel [22,23,24,25] of drugs within a single assay and/or they have not enough sensitivity [26,27,28,29]: these features are pivotal in the current TDM setting. In fact, the addition of TAF to the panel of NRTIs led to the need of higher sensitivity, since the expected concentrations of TFV (the active and toxic compound) in plasma are strongly reduced [12,13,30]. On the other hand, except for lamivudine (3TC) or zidovudine (also known as azidothymidine, AZT), the current NRTIs backbones consist in the combination of two drugs: most often, TAF with emtricitabine (FTC) or 3TC with abacavir (ABV). This combined therapeutic approach requires simultaneous quantification methods in order to manage an effective TDM service. Therefore, in this work we describe a fully validated and partially automated UHPLC-MS/MS method for the simultaneous quantification of the currently available NRTIs, including TFV, FTC, 3TC, ABV, AZT and the prodrug TAF, in plasma from PLWH and its application in the context of a clinical study.

## 2. Results and Discussion

### 2.1. Method Development and Preliminary Experiments

Some preliminary experiments were conducted in order to adapt previously developed methods to this analysis, including protein precipitation with acetonitrile (ACN) added with different percentages of formic acid in plasma samples with different anticoagulants. Since higher recovery for TFV was observed in sodium citrate plasma, probably due to its chelating activity on bivalent cations (which could potentially favor TFV co-precipitation), sodium citrate was added to all samples, in order to obtain similar recoveries both in sodium citrate and lithium heparin plasma.

**Figure 1:**
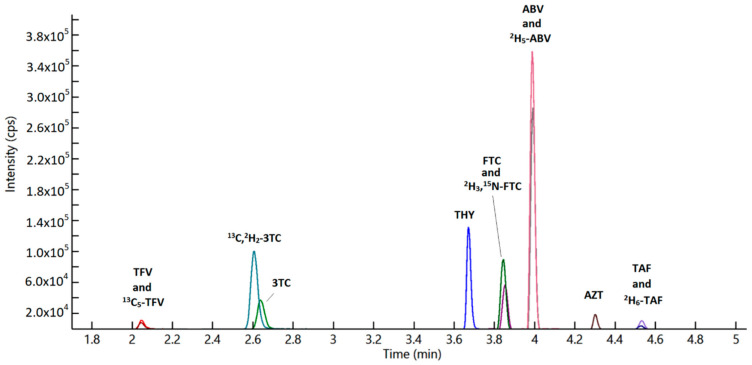
Overlaid chromatogram reporting all the considered drugs in this method in standard 5 (medium level in the standard curve).

Furthermore, the optimal recovery (REC) and matrix effect (ME) were obtained by using 7% (*v*/*v*) formic acid in acetonitrile as protein precipitation medium. Chromatographic separation was achieved in 5 min (Figure 1), but at least 1 min of column washing and 2 min of re-equilibration resulted necessary in order to increase column lifespan and performance on a long period (more than 3000 injections).

First, all the drugs were investigated for significant and reproducible ME among 6 different matrix lots with 2 different anticoagulants, and the performance of analogue internal standard (IS, thymidine (THY)) was evaluated in terms of Internal Standard-normalized ME (IS-nME) and IS-nREC.

This showed sub-optimal performance for TFV, 3TC, ABV, FTC and TAF (RSD for IS-nME or IS-nREC higher than 10%). Therefore, stable-isotope-linked ISs (SIL-IS) were first introduced for these drugs (depicted in Figure S1). Conversely, AZT showed an acceptable IS-nME using THY as IS, therefore THY was finally selected for method validation. ^2^H_6_-TFV was initially adopted as SIL-IS for TFV: nevertheless, this was associated to a slight difference in retention time (RT, 0.05 min before TFV) and to a significant interference from isobaric matrix components. Therefore, ^13^C_5_-TFV was finally selected as the most appropriate IS for TFV, yielding excellent performance.

### 2.2. Calibration Curve and Dilution Integrity

During method validation, TFV, ABV, AZT and TAF exhibited linear calibration curves, while quadratic models were used for 3TC and FTC. Mean coefficient of determination (r^2^) of all calibration curves for all drugs ranged from 0.996 to 0.998 (Table S1), confirming the optimal fitting to the calibration models. The back-calculated accuracies of the standard points of the calibration curve are reported in Table S2. Samples over the highest standard were successfully quantified after 1:2 (vol:vol) dilution with blank plasma, resulting in mean inaccuracy (percentage of deviation from the nominal value) and imprecision (relative standard deviation, RSD%) lower than 15%.

### 2.3. Specificity and Selectivity

The assay did not show any significant interference with other potentially concomitant drugs.

**Figure 2:**
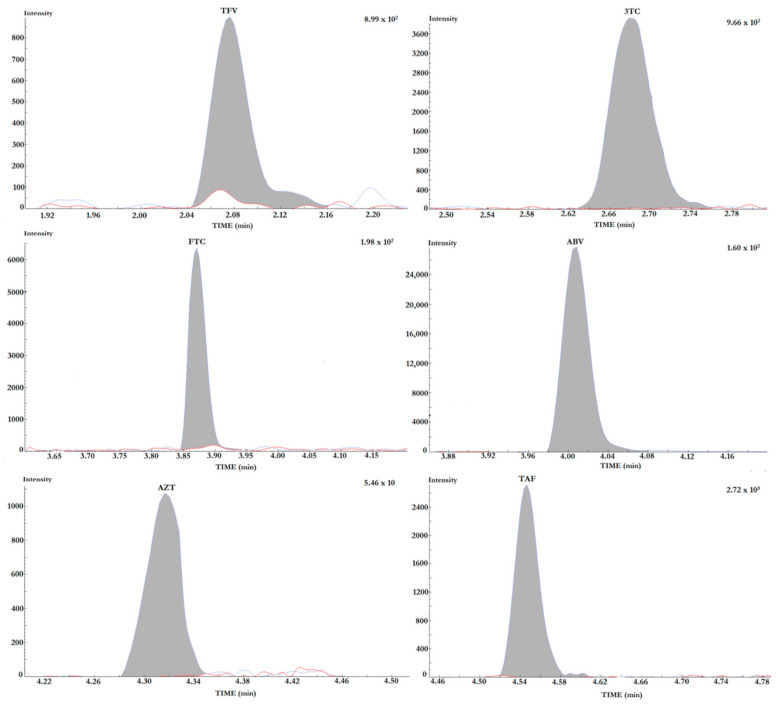
Overlaid chromatograms of each analyte quantification transition at the LLOQ (grey filled peaks) and in blank plasma (white filled). Extremely high signal-to-noise ratios were observed at the LLOQ for 3TC, ABV and AZT.

The RT of NRTIs were summarized in Table 1. The blank plasma samples did not yield any significant “noise” due to endogenous component at the analytes’ RTs (Figure 2).

### 2.4. Accuracy and Precision

Six validation sessions (six different days) have been performed in order to evaluate the accuracy and intra-day and inter-day precision at the three quality controls (QCs) concentrations (and at the LLOQ).

The validation results in terms of accuracy and precision are listed in Table 2. Both the RSD% and biases were below 15%, in compliance with FDA and EMA guidelines, except for the LLOQ, which showed acceptable bias (between −15.4% and +17.0%, Table S1) and RSD (between 16.3 and 17.5%).

### 2.5. Lower Limit of Quantification (LLOQ) and Limit of Detection (LOD)

The determination of the limit of detection (LOD) was carried out by executing serial dilutions of the lowest standard point of the calibration curve (STD1) for each drug. The lower limits of quantification and of detection are shown in Table 1. The LLOQ resulted at least equal to the STD1, as required by FDA guidelines. The overlaid chromatograms for each analyte at the LLOQ and in blank plasma are reported in Figure 2.

### 2.6. Recovery

Recovery data resulted highly reproducible for each compound and are summarized in Table 3. In lithium/heparin plasma AZT and TAF showed poor recovery: 68.7% and 74.0% respectively. Despite this, the variability in recovery resulted low and the overall performance of the assay was not affected. Moreover, recovery percentages resulted reasonably concordant between the target analytes and their IS compounds (showing low RSD for the IS-nREC).

### 2.7. Matrix Effect

The ME percentages resulted positive and quite high for several compounds (up to +49.8% for ABV), except for AZT, which showed ion suppression (−9.1%). These variations resulted reproducible between different plasma lots and anticoagulants (RSD always lower than 10%). Moreover, ME resulted efficiently compensated by the ISs, resulting in a low and highly reproducible (RSD < 10%) IS-nME for each compound (Table 3).

### 2.8. Carry-Over

The mean concentrations measured in blank plasma extracts injected immediately after samples containing high concentration of analytes was lower than LOD value. The mean IS area of blank plasma samples was lower than 1% of IS area of plasma samples containing IS.

These data showed the absence of significant carry-over.

### 2.9. Stability

No long-term stability at −80 or −20 °C, freezing and thawing or short-term autosampler stability evaluations were performed in this study since these data were extensively described in previous works [18,19,22,23,24,26].

On the other hand, the medium-term stability testing in plasma QC samples at 24–25 °C indicated that 3TC, FTC, ABV and AZT are stable for 14 days, showing a maximum degradation of 6.3% for AZT. No degradation was observed for other drugs (Table 4).

Conversely, TFV and TAF showed an extreme increase (+49%) and decrease (−100%), respectively, starting as soon as 2 days after thawing. This phenomenon was interpreted as a conversion of TAF to TFV, probably underlying the presence in plasma of low concentrations of hydrolases capable of metabolizing TAF [31,32]. The hypothesis was confirmed by retesting TFV stability in plasma residuals from samples which did not contain TAF at day 0, showing a slight decrease in TFV concentrations after 10 days (−19%). In this setting, daily TFV degradation rate was as low as 1.2% (equation: stability = 0.012 * days + 1.035). Further testing of a TAF solution prepared in water and kept at room temperature for 2 days did not show any conversion to TFV, suggesting the involvement of plasma enzyme in this conversion. Interpreting data from stability in QC samples in light of this observation, additional +60% of TFV would be expected from conversion of TAF to TFV which, combined with −16.8% TFV degradation, would clearly explain the observed deviation (+49%). This indicated that the only major concern for analytes’ stability within 1 week when kept at room temperature is the ex-vivo conversion of TAF to TFV.

### 2.10. Automation

Results from 3 sessions with 5 QC replicates extracted through the manual and the automated protocol confirmed RSD percentages within 6% through the automated protocol (comparable with validation data obtained manually) and maximum bias of +4.2% (for TFV) between the automated protocol and the manual one. Mean bias for the automated protocol compared with nominal values were within 5% for all the considered analytes, resulting comparable with the manual one. The automated protocol resulted in time savings of nearly 0.5 h for the procedure and 1.5 h for the operator.

### 2.11. Testing of Patients’ Samples

The new method has been tested on 32 samples from 16 PLWH which switched their treatment from elvitegravir/cobicistat/FTC/TAF (ELV/COBI/FTC/TAF, 150/150/200/10 mg q24 h) to bictegravir/FTC/TAF (BIC/FTC/TAF, 50/200/25 mg q24 h), at the pharmacological steady state (before and 1 month after the switch). All samples were successfully quantified for each drug, reporting concentrations within the calibration ranges. TAF was detected and quantified in 2 samples (32 and 396 ng/mL), together with abnormally high FTC concentrations (2330 and 722 ng/mL, respectively). This was interpreted as a good marker of inappropriate sampling time (e.g., C_max_ instead of C_trough_), since TAF half-life is estimated around 0.5–0.75 h in plasma [33], therefore its trough concentration should be always undetectable. Excluding the 2 patients with detectable TAF plasma concentrations, median TFV concentrations were 11.4 ng/mL (IQR 8.6–15.7) and 12.0 ng/mL (InterQuartile range, IQR 8.5–15.0) before and after the switch, resulting comparable (*p* = 0.523) despite the 2.5-fold increase in TAF dose. Similarly, FTC concentrations showed a not statistically significant reduction after the switch (*p* = 0.534), with median values changing from 230 ng/mL (IQR 131–280) to 172 ng/mL (IQR 97–240). Further application on 44 incurred samples containing 3TC, ABV or AZT for routine TDM applications resulted in median concentrations of 191 ng/mL (n = 40; IQR 109–406 ng/mL) for 3TC, 33 ng/mL (n = 10; IQR 23–87 ng/mL) for ABV and 151 ng/mL (n = 4; IQR 73–211 ng/mL) for AZT.

In order to evaluate incurred samples reanalysis, a fraction of these patients’ samples has been re-analyzed, showing acceptable bias: 5.1% for TFV (n = 20), 4.9% for 3TC (n = 22), 7.5% for FTC (n = 20), 2.8% for ABV (n = 7), 5.1% for AZT (n = 2) and 5.8% for TAF (n = 20).

## 3. Materials and Methods

### 3.1. Chemicals

Pure reference standard powders of TFV, 3TC, FTC, ABV and TAF (chemical structures depicted in Figure S2) were purchased from Clinisciences (Milan, Italy), all with a minimum purity of 98%; AZT was purchased from Sigma Aldrich (purity 99.5%). SIL-IS included racemic ^13^C_5_-TFV (purity 97.13%, isotopic purity 98.9%), ^13^C, ^2^H_2_-3TC (purity 96%, isotopic purity 99.2%) and were purchased from Toronto Research Chemicals (Toronto, Canada); ^2^H_5_-ABV (purity 99.23%, isotopic purity 99%), ^2^H_6_-TAF (purity 98.7%, isotopic purity 98%) and ^2^H_3_,^15^N-FTC (purity 100%, isotopic purity 98%) were purchased from Alsachim (Illkirch Graffenstaden, France). HPLC grade acetonitrile and methanol were purchased from VWR International (Radnor, PA, USA). HPLC grade water was produced with Milli-DI system coupled with a Synergy 185 system by Millipore (Milan, Italy). Formic acid and dimethyl-sulfoxide (DMSO) were purchased from Sigma-Aldrich (Milan, Italy) and Sodium citrate monobasic (>99.5%) was obtained from Sigma-Aldrich (Milan, Italy). Blank plasma from healthy donors was kindly supplied by the Blood Bank of the “Città della Salute e della Scienza” of Turin.

### 3.2. Stock Solutions, Standards and Quality Controls

All the pure powders were dissolved in DMSO to obtain stock solutions of 1 mg/mL of each drug (corrected by purity), which were stored at −20 °C until use, within 3 months.

Then, the stock solutions were used to independently spike blank plasma from healthy donors to obtain the highest calibration standard (STD 9) and 3 different quality control samples (QCs): high medium and low (QC H, M and L, respectively). All the lower STD samples (STD from 1 to 8) were obtained at each analytical session by serial dilution (1:1, v:v) of the STD 9 with blank plasma. Calibration ranges and QCs concentrations are summarized in Table 1 and Table 2 respectively. The STD concentrations for TFV and TAF were, as follows: 1.6 ng/mL, 3.1 ng/mL, 6.3 ng/mL, 12.5 ng/mL, 25 ng/mL, 50 ng/mL, 100 ng/mL, 200 ng/mL and 400 ng/mL for STDs 1 to 9, respectively. Conversely, STD concentrations for 3TC, FTC, AZT and ABV were 9.8, 19.5, 39.1, 78.2, 156.2, 312.5, 625, 1250 and 2500 ng/mL for STDs 1 to 9, respectively. The IS working solution was prepared by diluting ^13^C_5_-TFV and ^2^H_6_-TAF at 50 ng/mL, while ^13^C,^2^H_2_-3TC, ^2^H_5_-ABV and ^2^H_3_,^15^N-FTC were diluted at 500 ng/mL in water:methanol 50:50 (v:v), aiming at a concentration in the middle of the calibration range. All the solutions are already known to be stable at least for 2–3 month at −80 °C [22,23].

### 3.3. Method Development and Preliminary Experiments

Some preliminary experiments were conducted in order to adapt previously developed methods to this analysis. The acidic nature of TFV, which includes a phosphonic group, suggested the need to perform protein precipitation in acidic conditions. Therefore, increasing percentages of formic acid in acetonitrile (0.1%, 1%, 5%, 7% and 10% vol) were tested for the analytes’ REC and ME. Similarly, the effect of different anticoagulants (lithium/heparin and sodium citrate) was tested for the impact on analytes REC and ME. Moreover, several trials were performed to obtain the optimal internal standardization, using the evaluation of the IS-nME and IS-nREC as a marker of IS goodness.

The results of preliminary optimization are summarized in results section. After the completion of the preliminary experiments and optimization of the conditions for sample preparation, the method was fully validated on the basis EMA and FDA guidelines requirements, following the same validation process described in previous works [34].

### 3.4. Chromatographic Conditions

The chromatographic system was a LX50 UHPLC (Perkin Elmer), composed of an Integrity^®^ autosampler, a SPH1299^®^ Dual UHPLC Pump and a Mistral^®^ column oven. The chromatographic separation was performed using an Acquity^®^ UPLC HSS T3 column, 2.1 × 150 mm, 1.8 μm (Waters, Milan, Italy) at 40 °C. The temperature of the sample manager was set at 15 °C.

The flow rate was settled at 0.4 mL/min, with a gradient of two mobile phases (MP): MP-A (0.05% *v*/*v* formic acid in HPLC grade water) and MP-B (0.05% formic acid *v*/*v* in HPLC grade acetonitrile). Briefly, the chromatographic gradient started with 2% MP-B up to 0.2 min, then it was increased to 3% at 0.3 min and held at the same percentage up to 1.75 min; a linear increase to 12% MP-B was applied up to 2.4 min and, finally, further increased to 95% MP-B at 5 min. The column was washed for 1 min at 0.45 mL/min and then re-equilibrated to 2% MP-B up to the end of analysis. The total runtime was 8 min. water:methanol 95:5 vol:vol was used as weak washing solution, while water:acetonitrile 30:70 vol:vol was adopted as strong-washing solution. Two strong washing and 3 weak washing cycles (250 µL each) were applied, sequentially, after each injection.

### 3.5. Mass Spectrometry Conditions

Tandem mass spectrometry detection was carried out with a QSight^®^ 220 (Perkin Elmer, Milan, Italy) tandem mass spectrometer, with an electrospray ionization (ESI) interface. The ESI source was set in positive ionization mode (ESI+) for all drugs, except for AZT and its IS, THY, which were analyzed by negative ionization (ESI-). “Zero-Air” (Dry air) was used as nebulizing and heating gas, while nitrogen was used as Drying and Collision gas: both these gasses were produced at high purity (>99.9%) with a Cinel Zefiro Combined (Cinel, Vigonza, Italy).

Optimization of the MS conditions has been performed by single direct infusion of reference standards of each drug (100 ng/mL in a solution composed of MP-A:MP-B 50:50) at 10 μL/min into the mass spectrometer, combined with the flow of 50:50 A:B from the analytical column.

General mass parameters for positive ionization were: electrospray voltage 5.0 kV; source temperature 350 °C; nebulizing gas flow 350 L/h; heating gas flow 350 L/h; drying gas flow 130 L/h; Heated Surface Induced Desolvation (HSID) temperature 300 °C. Negative ionization (for AZT and THY) was performed at −4.0 kV, since they showed very low signals by positive electrospray ionization. Two mass transitions yielding the highest sensitivity were selected for all drugs except for ABV and 3TC and quantification was performed using multiple reaction monitoring (MRM) of the transitions reported in Table 1. For the analytes exhibiting two transitions, the first was used to quantify (quantification trace) whereas the second was used to confirm peak identity (secondary ion trace).

### 3.6. STDs, QCs and Patients’ Samples Extraction

After thawing at room temperature, each sample was treated as follows: 50 µL of IS working solution, 25 µL of sodium citrate solution and 925 µL of a precipitant solution (ACN 7% formic acid) were added to a volume of 100 µL of samples, standards and QC, and then, vortex-mixing for at least 10 s. Subsequently, all samples were centrifuged at 21,000× *g* for 10 min, without brake, at 4 °C. The supernatants (1 mL) were then transferred in glass shots and dried in a vacuum centrifuge at 50 °C for approximately 1.5 h. Finally, the dry extracts were dissolved with 0.8 mL of water, transferred in bulk vials and 4 µL were injected in the chromatographic system.

### 3.7. Specificity and Selectivity

Interference from endogenous compounds was investigated by analysis of six different blank samples. Potential interference by other concomitant drugs was also evaluated by spiking QC samples with them. These included antitubercular drugs as ethambutol, isoniazid, pyrazinamide, rifampicin and rifabutin, antibiotic drugs such as ceftriaxone, ceftobiprole, moxifloxacin, levofloxacin and linezolid and antifungal agents such as isavuconazole, fluconazole, posaconazole, voriconazole and itraconazole. An “interfering drug” has been considered as a molecule exhibiting an observable ion suppression/enhancement or cross-talk with any of the target analytes.

### 3.8. Accuracy, Precision, Calibration and Limit of Quantification

Intra-day and inter-day precision and accuracy were determined by analyzing three different QCs concentration (plus the LLOQ) in 6 different validation sessions. Accuracy was calculated as the ratio between the mean analytical result and the nominal concentration. Inter-day and intra-day imprecision were expressed as the relative standard deviation (RSD) at each QC concentration. Intra-day imprecision was evaluated by analyzing the three different QC samples (and at the LLOQ) in 5 replicates in the same analytical session.

Calibration curves were processed by analytes peak area normalized by areas of IS compounds.

The fitting to the calibration model was evaluated, for each drug up to STD9. Linear regression models with 1/x weighing were used for TFV, ABV, AZT and TAF, while quadratic models with 1/x weighing were used for 3TC and FTC; dilution integrity was tested by analyzing in 5 replicates samples spiked at drugs concentrations twice higher than STD 9 (the ULOQ), after a 3-fold dilution. The limit of detection (LOD) was defined as the plasma concentration that yielded a signal-to-noise ratio of 3:1. Percent deviation from the nominal concentration (measure of accuracy) and relative standard deviation (measure of precision) of the plasma concentration considered as the lower limit of quantification (LLOQ) had to be <20%, and it was set as the lowest calibration standard, as suggested by FDA and EMA guidelines [35,36].

### 3.9. Recovery

Recovery from plasma was assessed by comparing the peak areas obtained from multiple analyses of QC samples (high, medium and low) with the peak areas from the injection of blank extracts spiked with all the analytes after the extraction (post-extraction addition samples) at the same QCs concentrations, representing 100% recovery.

### 3.10. Stability

The long- and short-term stability data at −20 and −80 °C, as well as freezing and thawing stability, were already extensively described in previously published works [18,19,20,21,22,23]. Therefore, in this context, a medium-term stability study was conducted up to 14 days at room temperature (24–25 °C) on QC samples (H, M and L), in order to evaluate the feasibility of samples’ shipment without refrigeration. The chosen timings included 2, 4, 7, 10, 12 and 14 days. The percentage of stability was calculated as the ratio between analytes concentrations found in QC samples kept at room temperature and at −20°C, respectively. For TFV, FTC and TAF, stability evaluation was further performed on 2 spared samples corresponding to trough concentrations, comparing the results to those obtained at day 0.

### 3.11. Matrix Effect

The “matrix effect” (ME) was investigated on six lots of blank plasma with different anticoagulants (lithium heparin and sodium citrate), as suggested by the guidelines. Peak areas from blank extracts spiked with all analytes at three QC concentrations (post-extraction spiked samples) were compared with peak areas from standard solutions (prepared in pure water) spiked with all the analytes at the same concentration, as previously described [37]. The ME was calculated as the percentage of deviation of the peak areas obtained in presence of the matrix with the ones obtained from the standard solutions. Moreover, in order to estimate the capability of IS compounds of correcting ME associated variability, the “IS-normalized ME” (IS-nME) was calculated, as previously reported and suggested in EMA guidelines [35,38].

### 3.12. Carry-Over 

Carry-over was investigated in triple replicates by injecting extracted blank plasma samples immediately after samples containing target analytes at two-fold higher concentration than STD 9. A value ≤20% of the lower limit of quantification (LLOQ) and a value ≤5% for IS were considered as absence of significant carry-over.

### 3.13. Automation

The manually validated protocol was tested for its performance after adaptation for use on a Janus^®^ G3 (Perkin Elmer, Milan, Italy) robotic platform for liquid handling. This platform is equipped with an 8 line pipette with adjustable span, in order to allow the use of any kind of tubes or plates, and conductive tips, allowing liquid sensing. The overall extraction protocol was identical to the manual one, with only minor adaptations. The liquid handler performed the spiking with IS working solution, then the serial STD 9 dilutions were performed, in order to obtain the lower STD samples. One hundred microliters of STDs, QC and patients’ samples are then transferred to 1.5 mL tubes, already containing the IS solution. The samples were then added with 25 µL of sodium citrate solution and 925 µL of ACN acidified with 7% of formic acid. After protein precipitation, 950 µL were recovered, setting the aspiration speed at 75 µL/s at 1.5 mm under liquid surface (set in “liquid tracking” mode, in order to follow the supernatant level, constantly under its surface), and transferred into the glass shots. After the drying step, the automated platform reconstituted the dry extracts with 0.8 mL of water. Finally, the samples were centrifuged again in the vacuum centrifuge for 3 min, in order to obtain the complete precipitation of residual solid particles. A volume of 700 µL of supernatant was aspirated at 70 µL/s at 0.5 mm under the liquid surface (with “liquid tracking”), and then transferred into the vials for analysis.

### 3.14. Clinical Application and Statistical Analysis

This method was further applied both for routine TDM purpose and in the context of the observational clinical study “*Appropriatezza farmacologica della terapia antinfettiva*” (ethical approval n. 0040388 23/04/2020), which aimed at describing antimicrobial drug concentrations in the context of treatment switches commonly applied in the current clinical practice. In this case, a subset of PLWH switching from ELV/COBI/FTC/TAF 150/150/200/10 mg q24 h cART regimen to BIC/FTC/TAF 50/200/25 mg q24 h were enrolled. The study has been conducted in compliance with the Declaration of Helsinki and all patients gave informed consent before enrolment.

Blood sampling was performed at the end of dosing interval (C_trough_) with lithium/heparin vacutainers, the morning before the treatment switch and 1 month after the switch, at the pharmacological steady state. The concentrations of FTC, TFV and TAF were measured in plasma samples obtained after centrifugation at 1400× *g* for 10 min at 4 °C. The results were then compared through non-parametric Wilcoxon paired rank test, by using SPSS software (ver. 26.0, IBM, Armonk, NY, USA). All concentration data were described as medians and IQR. Chromatographic and mass spectrometry data were processed through Simplicity^®^ 3Q (Perkin Elmer, Milan, Italy) software.

## 4. Conclusions

In the last years, the application of TDM is becoming a standard practice for several therapies, among which cART is one of the most relevant. In this work, a validated UHPLC-MS/MS method has been described, characterized by high sensitivity, reliability and cheapness. Compared to previously described methods [22,23,24,26,27,28], this is the first capable to quantify TFV at the very low concentrations with its pro-drug TAF, together with all the other currently used anti-HIV NRTIs, with a simple protein precipitation protocol. Moreover, this high sensitivity was achieved with a consistent reduction of sample volumes (100 µL) compared with our previous method [29], potentially allowing its use coupled with microsampling strategies or with spared plasma samples. Furthermore, the capability to be performed both manually or with an automated platform further widens the application range, allowing a potential increase in the repeatability and reproducibility of the sample preparation and a reduction of the operative time, as well as the infective risk. Another important achievement is the capability to detect and quantify the prodrug TAF, providing useful information about the sampling timing (real trough concentrations vs wrong timings) and/or their conversion, useful in case of intensive PK sampling for the evaluation of the whole area under the concentration-time curve.

Furthermore, the multiplexed approach allows a simpler TDM routine management and results also useful in order to control and detect eventual erroneous TDM requests (TDM required for the wrong drug), which can be possible, particularly in case of treatment switches.

The described method was based on a thorough selection of the optimal precipitating solution in order to achieve the highest possible recovery of TFV which, with the introduction of TAF, is the compound with the lowest expected concentrations in human plasma [12,33,39]. Slightly turbid extracts were observed after drying due to the presence residual solid residuals, which were efficiently discarded by a rapid centrifugation: this measure resulted in a long column lifetime and a contained variability in the observed IS-nME and IS-nREC, also taking into account different anticoagulants. This is, in our knowledge, the first study to evaluate these parameters across different anticoagulants, showing their potential impact on analytical performance and verifying the robustness of the analytical strategies (use of isotope-labeled ISs, optimization of extraction procedure, etc.) in copying with this issue.

The application of this method on a clinical study evidenced comparable TFV plasma concentrations in patients switching from a 10 mg dose with COBI-boosted elvitegravir to a 25 mg dose with bictegravir. This confirms the appropriateness of current dosing [40] in PK terms, in accordance with the capability of COBI of increasing the bioavailability of TAF [41]: in fact, TAF is a P-gp and BCRP substrate and, therefore, efflux transporters inhibitors such as COBI or RTV are capable to reduce its elimination. Nevertheless, while this is confirmed for TFV exposure in plasma, recent data suggest lower TFV-diphosphate (the active intracellular TFV metabolite) concentrations within PBMC and lymphoid tissues when given with COBI [42]. Finally, the evidence of detectable TAF concentrations only in samples showing abnormally high FTC concentrations seems to indicate wrong timing for drug intake or PK sampling. In fact, according to previous studies TAF is rapidly metabolized, being undetectable within 8 h (12 h in case of severe renal impairment) from the last intake: therefore, high TAF concentrations may be interpreted as an interesting marker of “white-coat” adherence to the treatment. This evidence, together with the described method, will be useful in the near future as a guidance for optimal TDM performance and for PK studies, potentially improving the management of cART in patients who are treated with a NRTI backbone.

## Figures and Tables

**Table 1:** For each drugs are reported, in order: retention time (RT), the concentration in the highest standard point of the calibration curve (STD 9 to STD1/LLOQ), LOD, dwell times and mass transitions (parent ions, first and second daughter ions), with the corresponding entrance voltages and collision energies. All concentration data are referred to the initial plasma sample.

DRUGs	RT (min)	STD 9 (ULOQ) (ng/mL)	Calibration Range (ng/mL)	LLOQ (ng/mL)	LOD (ng/mL)	[M + H]^+^ (m/z)	Dwell Time (ms)	Entrance Voltage (V)	FIRST Trace (m/z)	Collision Energy First Ion Trace (eV)	SECOND Trace (m/z)	Collision Energy Second Ion Trace (eV)
^13^C_5_-TFV	2.05	-	-	-	-	293.1	25	30	181.1	−30	164.1	−45
TFV	2.05	400	1.6–400	1.6	<0.8	288.1	25	30	176.1	−30	159.1	−45
^13^C_1_-^2^H_2_-3TC	2.62	-	-	-	-	233.1	25	10	113.1	−42	-	-
3TC	2.66	2500	9.8–2500	9.8	<5.0	230.1	25	10	112.1	−42	-	-
THY *	3.66	-	-	-	-	241.1 *	25	−26	42.0	74	151.0	15
^2^H_3_-^15^N-FTC	3.87	-	-	-	-	252.0	25	15	132.1	−37	114.0	−65
FTC	3.88	2500	1.6–400	9.8	<5.0	248.0	25	15	130.1	−37	113.0	−65
^2^H_5_-ABV	4.00	-	-	-	-	292.2	25	24	196.1	−37	-	-
ABV	4.01	2500	9.8–2500	9.8	<5.0	287.2	25	24	191.1	−37	-	-
AZT *	4.32	2500	9.8–2500	9.8	<5.0	266.1 *	25	−17	223.0	15	193.0	19
^2^H_6_-TAF	4.55	-	-	-	-	483.2	25	40	271.0	−42	346.1	−33
TAF	4.55	400	1.6–400	1.6	<0.8	477.2	25	40	270.0	−42	345.1	−33

* Negative Ionization [M − H]^−.^

**Table 2:** Summary of quality controls (QCs) concentration. accuracy. intra-day and inter-day precision (relative standard deviation. RSD%) for all drugs.

DRUG	QC High				QC Medium				QC Low				OVERALL		
	Conc. ng/mL	Acc. %	Precision RSD%		Conc. ng/mL	Acc. %	Precision RSD%		Conc. ng/mL	Acc. %	Precision RSD%		Acc. %	RSD Intra-Day %	RSD Inter-Day %
			Intra-Day %	Inter-Day %			Intra-Day %	Inter-Day %			Intra-Day %	Inter-Day %			
TFV	320	102.4	3.4	3.5	20	99.2	2.4	4.4	4	100.9	6.7	6.3	100.9	4.5	4.7
3TC	2000	104.0	3.4	1.9	100	106.1	1.0	1.5	20	106.8	1.6	5.2	105.6	2.4	4.7
FTC	2000	104.6	3.2	5.8	100	109.6	1.5	6.9	20	94.3	4.4	3.0	102.9	7.1	6.5
ABV	2000	104.3	2.6	3.2	100	106.9	5.7	2.6	20	95.8	4.1	3.7	102.3	6.3	3.1
AZT	2000	106.5	2.8	3.5	100	101.6	8.0	3.9	20	100.2	7.3	4.6	102.4	6.5	4.0
TAF	320	107.6	2.9	5.2	20	101.0	6.2	5.4	4	98.4	9.7	6.3	102.3	7.2	5.6

**Table 3:** Overall summary of the observed mean recovery (REC) and matrix effect (ME) data for all drugs in plasma samples with lithium heparin and sodium citrate as anticoagulants. respectively. IS-nREC = recovery normalized by the IS; IS-nME = Matrix effect normalized by the IS; RSD = Relative Standard Deviation.

**Lithium/Heparin Samples (n = 6)**				
**Drug**	**Mean REC % (RSD %)**	**Mean IS-nREC % (RSD %)**	**Mean ME % (RSD %)**	**Mean IS-nME %** **(RSD %)**
TFV	82.4 (5.6)	106.9 (0.3)	+19.6 (4.4)	−1.6 (5.1)
3TC	103.2 (2.7)	103.7 (1.4)	+23.5 (1.1)	−2.2 (1.3)
FTC	103.2 (4.0)	104.2 (1.9)	+25.8 (2.4)	−4.1 (2.3)
ABV	91.2 (1.7)	110.3 (0.4)	+49.8 (2.8)	−1.2 (2.5)
AZT	68.7 (7.0)	72.2 (6.2)	−9.4 (1.3)	−2.2 (1.5)
TAF	74.0 (2.0)	96.2 (5.1)	+17.5 (3.4)	+4.5 (3.4)
**Sodium Citrate Samples (n = 6)**				
**Drug**	**Mean Rec. % (RSD %)**	**Mean IS-nREC % (RSD %)**	**Mean ME% (RSD %)**	**Mean IS-nME %** **(RSD %)**
TFV	94.1 (1.7)	101.1 (9.7)	+33.1 (6.2)	+8.3 (4.4)
3TC	110.5 (2.7)	105.9 (0.8)	+21.3 (1.5)	−1.6 (0.2)
FTC	107.2 (3.3)	105.0 (1.2)	+21.0 (2.1)	−3.3 (2.4)
ABV	90.1 (1.6)	105.8 (2.5)	+42.0 (3.2)	+1.8 (2.8)
AZT	71.3 (3.8)	79.7 (4.0)	−8.9 (2.2)	−2.5 (2.3)
TAF	92.3 (5.1)	101.2 (3.8)	+23.0 (2.1)	−2.9 (3.2)
**OVERALL**				
**Drug**	**Mean Rec. % (RSD %)**	**Mean IS-nREC % (RSD %)**	**Mean ME% (RSD %)**	**Mean IS-nME %** **(RSD %)**
TFV	88.2 (8.3)	104.0 (7.8)	+26.3 (6.2)	+3.4 (5.5)
3TC	106.8 (4.3)	104.8 (1.5)	+22.4 (1.0)	−1.9 (0.3)
FTC	105.2 (3.7)	104.6 (2.5)	+23.4 (2.3)	−3.7 (4.5)
ABV	90.6 (1.5)	108.1 (2.2)	+45.9 (3.1)	+0.3 (1.7)
AZT	70.0 (5.0)	78.4 (5.9)	−9.1 (0.3)	−2.3 (1.8)
TAF	83.1 (13.2)	98.7 (5.9)	+20.2 (2.6)	−0.8 (5.4)

**Table 4:** Summary of the analytes’ stability at room temperature. Percentages of stability have been calculated by direct comparison with frozen quality control samples (for stability in quality controls) and with the concentrations at day 0. for the evaluation in patients’ samples. The stability evaluation in patients’ samples was limited to 12 days due to low sample volume.

Room Temperature Stability in Quality Control Samples						
	2 Days	4 Days	7 Days	10 Days	12 Days	14 Days
TFV	120.9%	138.4%	156.8%	142.4%	148.2%	149.0%
3TC	111.1%	102.1%	106.0%	98.8%	102.0%	102.4%
ABV	110.6%	103.4%	106.0%	101.7%	101.8%	102.4%
FTC	111.9%	101.1%	103.2%	101.0%	97.8%	105.1%
TAF	53.8%	23.1%	8.2%	2.7%	7.0%	0.0%
AZT	93.0%	100.0%	102.0%	103.7%	89.3%	93.7%
**Room Temperature Stability in Patients Samples at the End of Dosing Interval (C_trough_)**						
TFV	99%	99%	109%	81%	89%	n.a.
FTC	90%	92%	99%	121%	97%	n.a.
TAF	n.d.	n.d.	n.d.	n.d.	n.d.	n.a.

n.a. = not applicable; n.d. = not detectable.

## Data Availability

Data will be provided on request.

## Supplementary Materials

The following are available online at https://www.mdpi.com/article/10.3390/ph14050460/s1, Figure S1: Chemical structures and labeling positions for the SIL-ISs; Figure S2: Chemical structures of the target drugs (based on structures on pubchem database (https://pubchem.ncbi.nlm.nih.gov/); Figure S3: Calibration plots for each analyte. Black dots represent standards, violet dots quality controls and orange dots unknown samples (orange dots higher than STD 9 represent high concentration chemical mix for system suitability); Table S1: Calibration parameters; Table S2: Back-calculated accuracy of the calibration standards.
