# Supplementary material for: Development and Validation of an Up-to-Date Highly Sensitive UHPLC-MS/MS Method for the Simultaneous Quantification of Current Anti-HIV Nucleoside Analogues in Human Plasma
Source: Pharmaceuticals (Basel). 2021 May 13;14(5):460. doi: 10.3390/ph14050460 (PMC8153023; doi:10.3390/ph14050460)
Supplement: Supplementary file 1 [file pharmaceuticals-14-00460-s001.zip › Supplementary materials - Calibration parameters.pdf]

Supplementary table S1: Mean observed calibration data for the each analyte. Y is the chromatographic response (Analyte/IS area ratio), while *x* is the analyte concentration; 1/*x* weighing was applied, in order to obtain optimal fitting to the lowest part of the calibration curves.

DRUGs	R^2	Equation
**TFV**	0.997	$y = 0.0410\,x + 0.0172$
**3TC**	0.998	$y = -(2.03\text{x}10^{-7})\,x^2 + 0.0032\,x + 0.0060$
**FTC**	0.997	$y = -(1.94\text{x}10^{-7})\,x^2 + 0.0037\,x + 0.0081$
**ABV**	0.996	$y = 0.0041\,x + 0.0108$
**AZT**	0.996	$y = 0.00073\,x + 0.00112$
**TAF**	0.997	$y = 0.1810\,x + 0.0851$

Supplementary table S2: Mean accuracy percentages of the back-calculated concentrations of calibration standards (STDs) during the validation sessions.

DRUGs	STD1 - LLOQ	STD2	STD3	STD4	STD5	STD6	STD7	STD8	STD9 - ULOQ
**TFV**	116.2	108.1	96.2	98.2	103.6	102.7	98.1	98.2	96.6
**3TC**	114.5	105.6	102.3	101.5	104.9	102.3	99.8	97.1	97.5
**FTC**	116.2	109.7	100.2	99.8	101.2	100.6	96.7	97.2	96.3
**ABV**	84.6	103.7	104.2	105.2	106.7	105.6	103.1	98.2	95.6
**AZT**	112.2	108.3	107.2	98.7	102.2	106.3	101.7	98.0	96.2
**TAF**	117.0	106.3	104.8	102.1	98.3	100.5	98.5	97.6	97.4
