# Supplementary figures and images for: Development and Validation of an Up-to-Date Highly Sensitive UHPLC-MS/MS Method for the Simultaneous Quantification of Current Anti-HIV Nucleoside Analogues in Human Plasma
Source: Pharmaceuticals (Basel). 2021 May 13;14(5):460. doi: 10.3390/ph14050460 (PMC8153023; doi:10.3390/ph14050460)

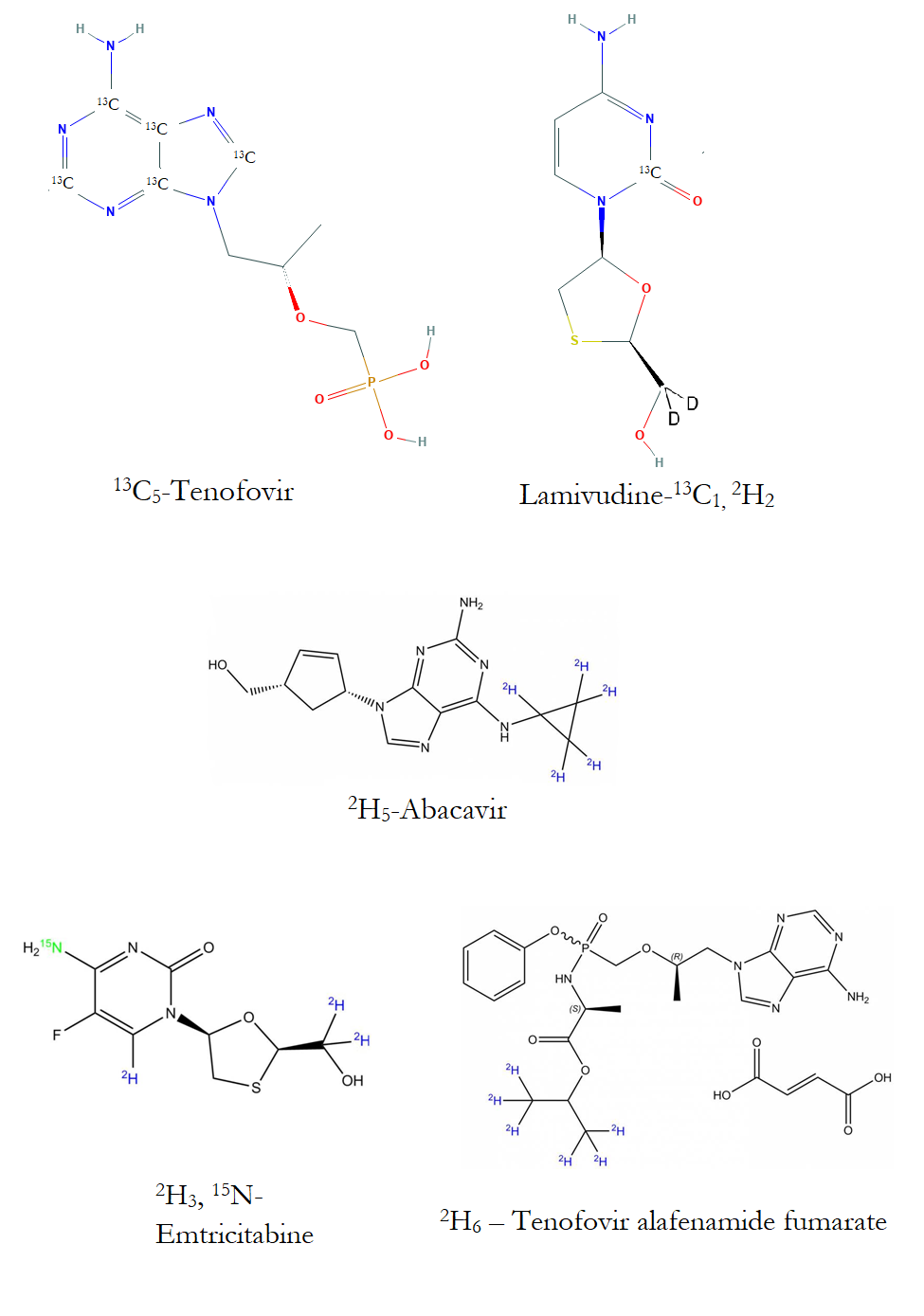

Supplement: Supplementary file 1 [file pharmaceuticals-14-00460-s001.zip › Figure S1.png]

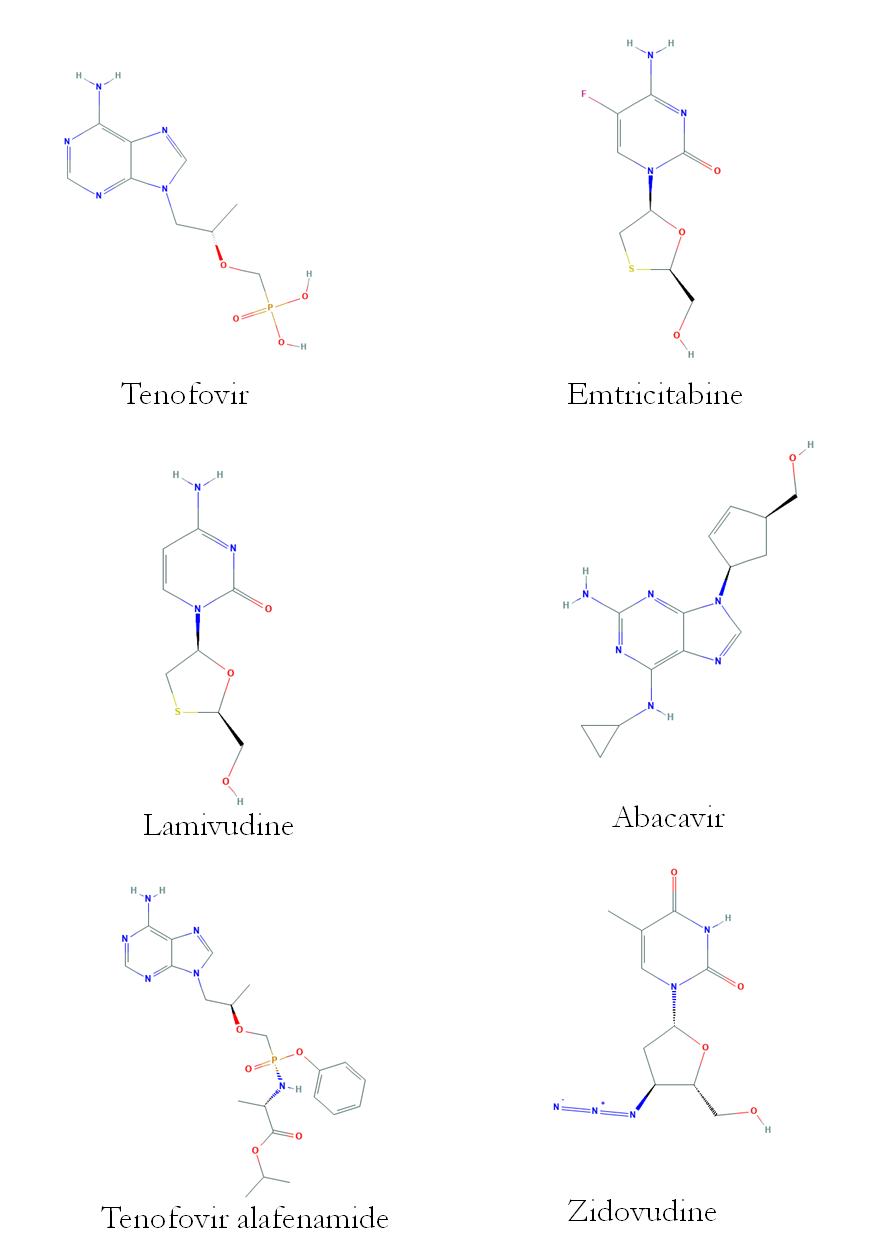

Supplement: Supplementary file 1 [file pharmaceuticals-14-00460-s001.zip › Figure S2.png]

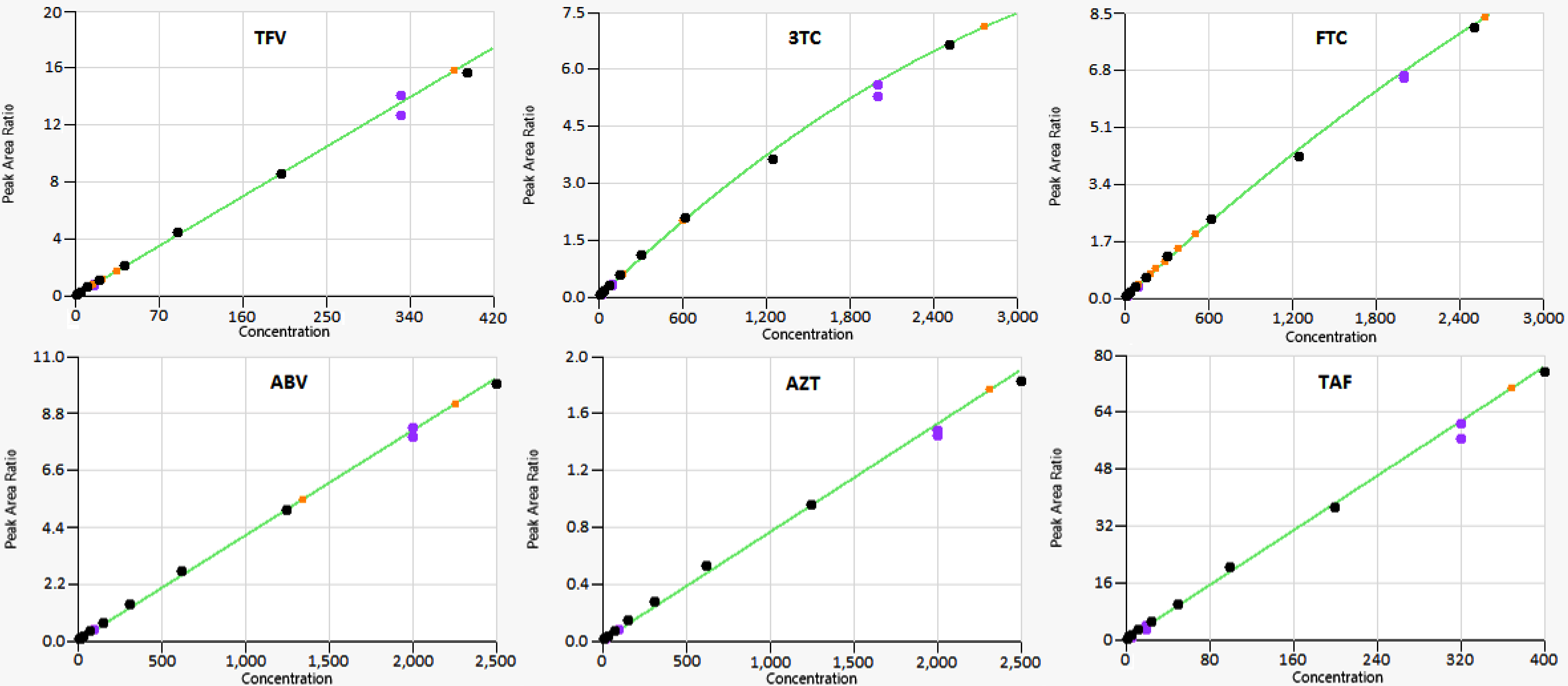

Supplement: Supplementary file 1 [file pharmaceuticals-14-00460-s001.zip › Figure S3.tif]
